# Management and outcomes of side effects with focus on anaemia in patients with hepatitis C genotype 1 infection: the telaprevir early access program in patients from Romania

**DOI:** 10.1186/1471-2334-13-S1-P59

**Published:** 2013-12-16

**Authors:** Adrian Streinu-Cercel, Anca Victorița Trifan, Florin Alexandru Căruntu, Ioan Sporea, Liliana Simona Gheorghe, Manuela Curescu, Mihai Mircea Diculescu, Mihai Voiculescu, Oliviu Pascu, Isabelle Lonjon-Domanec, Andrew Martin Hill, Sorin Rugină

**Affiliations:** 1 Carol Davila University of Medicine and Pharmacy, Bucharest, Romania; 2 National Institute for Infectious Diseases “Prof.Dr. Matei Balş”, Bucharest, Romania; 3 “Gr.T.Popa” University of Medicine and Pharmacy, Iaşi, Romania; 4 Institute of Gastroenterology and Hepatology, “St Spiridon” Emergency Hospital, Iaşi, Romania; 5 Victor Babeş University of Medicine and Pharmacy, Timişoara, Romania; 6 Center for Digestive Diseases and Liver Transplantation, Fundeni Clinical Institute, Bucharest, Romania; 7 Regional Institute of Gastroenterology and Hepatology, Cluj-Napoca, Romania; 8 Janssen Pharmaceuticals, Paris, France; 9 MetaVirology Ltd, London, UK; 10 Ovidius University, Constanța, Romania; 11 Clinical Hospital of Infectious Diseases, Constanța, Romania

## Background

Anaemia is a common adverse event during treatment for HCV infection. HEP3002 is an ongoing, open-label, early access program of telaprevir in 16 countries, for patients with genotype 1 hepatitis C with severe fibrosis or compensated cirrhosis. This analysis is of the data from the 209 Romanian patients, evaluated after 16 weeks of treatment.

## Method

Liver biopsy or non-invasive tests showing severe fibrosis or cirrhosis were required at entry. 209 patients from Romania were treated with telaprevir in combination with peginterferon alfa and ribavirin (PR) for 12 weeks, followed by PR for 12 or 36 weeks. Use of iron supplements, erythropoietin (EPO) and blood transfusions was permitted. Anaemia included the clinically significant adverse event terms of anaemia or haemoglobin (Hb) reduction. All analyses were on the Intent to Treat (ITT) population, using 16 week data.

## Results

Mean age was 52 years; 47% were male and 100% Caucasian, 59% had HCV RNA levels ≥800,000 IU/mL, 58%/42% had severe fibrosis/cirrhosis, and 2% had genotype 1a. Up to week 16, 57% of patients developed grade 1-4 anaemia, with 41% Grade 3-4 cases (3% grade 4); 72 patients (34%) dose-reduced ribavirin, and 1 (<1%) discontinued treatment for anaemia. Results are shown in Table 1.

**Table 1:** 

Type of anaemia	Definition	Total (n=209)
**Grade 1-2**	**Hb 9-10.9 or 2.5-4.4 g/dL decrease**	**33 (16%)**
**Grade 3-4**	**Hb <8.9 or >4.5 g/dL decrease**	**86 (41%)**
**D/C for anaemia**		**1 (<1%)**
**Anaemia as Serious AE**		**9 (4%)**

Up to week 16, 46% of patients developed grade 3 or 4 adverse events including 5 patients (2%) who developed grade 3 or 4 rash; 9% of patients had serious adverse events. Nine patients (4%) discontinued TVR due to adverse events, including 6 patients (3%) who discontinued due to rash. No deaths occurred during the study.

## Conclusion

In this telaprevir early access program for patients with severe fibrosis or compensated cirrhosis, Grade 3 or 4 anaemia was reported in 41% of patients, but discontinuation for anaemia was rare (<1%). Anaemia was principally managed by ribavirin dose reduction**.**

